# Water sanitation problem in Pakistan: A review on disease prevalence, strategies for treatment and prevention

**DOI:** 10.1016/j.amsu.2022.104709

**Published:** 2022-09-16

**Authors:** Khulud Qamar, Goodluck Nchasi, Hania Tul Mirha, Javeria Arif Siddiqui, Kainat Jahangir, Sean Kaisser Shaeen, Zarmina Islam, Mohammad Yasir Essar

**Affiliations:** a Faculty of Medicine, Dow University of Health Sciences, Pakistan; b Catholic University of Health and Allied Sciences, Tanzania; c CMH Lahore Medical College, Lahore, Pakistan; d Faculty of Medicine, Sindh Medical College, Jinnah Sindh Medical University, Pakistan; e Kabul University of Medical Sciences, Kabul, Afghanistan

**Keywords:** Water sanitation, Pakistan, Water crisis, Diseases, Water treatment

## Abstract

The global water crisis is expected to worsen if urgent action is not taken in making sustainable amends. This applies to Pakistan as the entire country suffers massively from poor water sanitation. Waterborne diseases are rising exponentially attributed to rise in microbial infestations, trace elements and drug toxicity in many water bodies of Pakistan. Treatment and prevention strategies must be implemented through national authorities and at the individual level. Awareness on use of clean water must be emphasized and proper administration on water management policies should be implemented. Immediate and active sustainability for water resources can ensure a safer future for Pakistan.

## Highlights

• Water Sanitation has been a problem all over Pakistan.
• The current situation has shown that most rural areas and some urban areas suffer due to the unclean water supply, which progresses in many diseases.
• This paper combines many methods of preventing waterborne diseases and treating the water bodies to make it free from contaminants.

## 1 Introduction

Water is one of the most vital necessities for survival, the World Health Organization (WHO) states water as a “primary tool for enhancing public health” as availability of water prevents many diseases and significantly improves hygiene [1]. Access to clean water is a right to all yet a privilege to only a few as globally more than 1.1 billion people face water scarcity and 2.4 million lack access to clean water [2]. Among the countries with insufficient water and sanitation problems, Pakistan has been ranked third according to the International Monetary Fund (IMF) as 2.1 million Pakistani individuals are deprived of safe water [3].

Pakistan's availability and quality of water is feared to present with many complications if not addressed urgently. Zhang et al. have stated that water shortage and cleanliness has critically affected many of the country's vital components such as the agricultural, environmental and societal systems [3]. There have also been many threats to Pakistan's public health due to water sanitation and hygiene problems as the risks for waterborne diseases exponentially increases. In 2017, 2.5 million deaths due to diarrhea were reported in Pakistan; 50% of the country's diseases and 40% of deaths occur due to consumption of contaminated water [4]. The water is contaminated with many pollutants such as fecal matter and microbes, metallic toxins, factorial and household waste, antibiotics, and other harmful drugs. The reasons of this water crisis in Pakistan include: climate change that impacts the yearly rainfall, poor development of water storage structures, and political influences [[3], [4]]. [[,^4^] [][3], [4][] Other causes include increased demand because of the mass population and industrial surge that shorten the water supply [3]. Pakistan is severely challenged at an economic level as poor water sanitization has costed around 343.7 billion PKR (1.5 billion USD) in 2019 [5]. Furthermore in 2016–17 with the collaboration of UNICEF, the cost of allocating the services for cleaner water had increased to 72 billion PKR after previously being 48 billion PKR [5]. Even these services were of not optimal quality and available to the entire country hence, it can be said that supplying sanitized water in Pakistan will require funding. It is predicted that if the following conditions are not improved, many of the current challenges such as poverty, prevalence of disease and economic instability will worsen multiple folds in the near future [6].

This paper aims to present the impacts due to lack of water sanitation in Pakistan, challenges faced by the country and disease outbreaks. It will also highlight further investigations required on this crisis and discuss recommendations for improving the water quality and accessibility in the country.

## 2 Methodology

For this paper, the databases Google Scholar and PubMed have been used, key words “Water Sanitation”, “Hygiene” and “Pakistan” were used. Additional searches were “Diseases”, “Water Contamination”, “Water Shortage”, “Waterborne diseases”, “Water purification techniques”, “WASH”. All article types i.e., observational, editorial, review studies respective to the literature search have been included in this narrative review. Literature that does not correspond to situations in Pakistan or diverts away from the key words are excluded as well as any duplicates.

## 3 Results

**Fig. 1:**
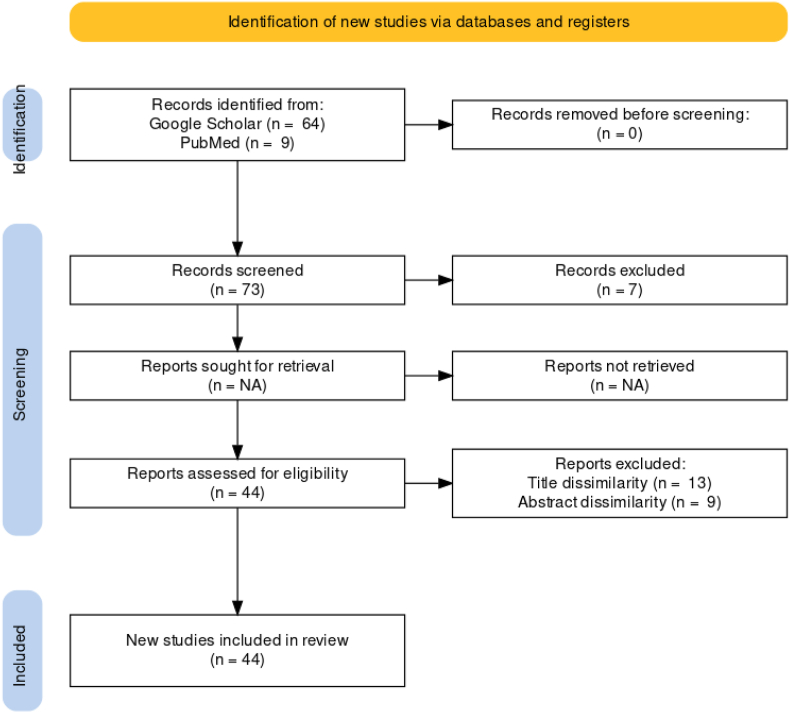
The studies shortlisted from the identification and screening process have been shown in the PRISMA flowchart below.

As relevant to the respective sections, 12 studies have been included regarding the status of water sanitation across the cities and provinces of Pakistan. Moreover, 17 studies have been included regarding the condition of water bodies, contaminants, and waterborne disease prevalence in Pakistan. Additionally, 7 mores studies have been included regarding the preventative techniques and treatment of water bodies, alongside this for recommendations 2 more studies have been included. A total of 44 reference articles have been included in this review including the introduction section with 4 additional studies. Identification, screening, and inclusion has been summarized on Fig. 1.

## 4 Current situation of water sanitation in Pakistan

Access to safe, quality drinking water is a fundamental right of life, and it is the responsibility of those in charge to ensure that this right is upheld. The distribution of safe drinking water in Pakistan is markedly skewed, with only 20% of the population having access to quality water [4]. As a result, the remaining population is left with substandard quality water to fulfil daily requirements, undoubtedly exposing them to many diseases and other toxic effects of contaminated water, aggravated by anthropogenic activities. The most common pathogen found in drinking water are coliforms [7]. Inadequate sanitation of this water results in diarrhea upon consumption, which is one of the major causes of death in children under the age of 5 in Pakistan [8]. A study on the Prevalence of Pathogenic Microorganisms in Drinking Water of Rawalpindi and Islamabad revealed that less than half the samples collected were satisfactory for drinking, while the remaining samples were unsatisfactory and riddled with pathogens and fecal contaminants [7]. Furthermore, a study on the Samanduri drain in Faisalabad revealed untreated wastewater discharge being the largest contributor of contamination, resulting in water quality being incredibly poor and unfit for drinking [9]. Moreover, a study into water quality across Pakistan highlighted the various contaminants and challenges of water sanitation. Water in Punjab was found to be majorly contaminated by microbiologic factors and arsenic, thus rendering the water unfit for use and a major health risk [10]. Similarly, water quality in Khyber Pakhtunkhwa was also poor, with majority being microbiologically contaminated and also majorly contaminated by iron [10].Water samples in Balochistan were also majorly contaminated by microbes and many nitrates, whilst water in Sindh was also found to be largely microbiologically contaminated [10]. Consuming inadequately sanitized water contaminated with pathogenic microbes poses major health risks, with approximately 80% of diseases in developing countries like Pakistan being waterborne [11]. The immense increase in prevalence of contaminated water can be attributed to the increase in population and therefore an increase in littering and disposal of household waste into water bodies [4,12]. This constant discharge has deteriorated the quality of surface water, has introduced toxic pollutants, and ultimately negatively impacting human health and the environment in the long term. Furthermore, the dumping of factory and agricultural waste into water bodies has contributed significantly to the contamination of water. The agricultural sector in Pakistan utilizes majority of the water, and the runoff generated coupled with the extensive use of fertilizers have increased contaminants like fluoride greatly [13].

In Pakistan, with the exponential increase in population and lack of fresh water sources, people are forced to rely on untreated ground water as their primary source, which is largely saline and does not meet WHO standards [14]. Over the years, the water availability has reduced by 3900 cubic meters per capita since 1951 to 2009. It is estimated to become as scarce as 700 cubic meters per capita in 2025 [15]. The severe shortage current water storage facilities and uncertainty of more projects working for Pakistan's drainage, sanitization and storage are reasons why water shortfall is believed to reach 32% in 2025 [15]. Pakistan is currently battling one of the most severe climate changes with the recent flooding, which too has further troubled water management.

With sewage pipes and the water sanitation pipes running alongside each other, leakage of these pipes causes contamination of the water, exposing users to a host of diseases [16]. For water to be deemed safe, it must be “free from components which may adversely affect the human health. Such components include minerals, organic substances and disease-causing microorganisms.“(17) An assessment of water quality, using the water quality index (WQI) in Sindh found that only 2.13% of the samples were excellent while 55.32% were classified as poor [12]. With no alternative source for drinking water, coupled with the demand for clean water greater than supply, majority of the population is forced to use this water. This has resulted in a cardinal impact on human health, with 30% of all diseases and 40% of all deaths due to poor water quality [17].

## 5 Diseases

Globally, one in three people do not have access to clean drinking water while more than 673 million people still practice open defecation thus predisposing them to waterborne diseases [18]. As of 2016–2017, only half of the population in urban sub-Saharan Africa had access clean drinking water and about half a billion deaths are caused by diarrheal diseases [19,20]. In rural Africa, there is still limited access to clean water due to underdeveloped sanitation systems hence this predisposes the people to many infections leading to chronic disability and death [21].

In Pakistan, waterborne diseases account for 80% of all diseases and 33% of deaths [4]. In addition, there is a high national expenditure of Pakistani rupee (PKR) 112 billion per annum due to hygiene related illness; it includes disease caused by unsafe water and poor sanitation [22].

Pakistan is blessed with many surface and ground water resources [10]. However, the increased technological development has led to the introduction of biological, chemical, and physical impurities in drinking water. Studies have shown cotton farms in Multan and Punjab province cause ground water contamination by pesticides, where 33% of samples exceed the maximum residue limit [23]. Moreover, Improper disposal of industrial waste has introduced metals such as copper and iron, a country-wide study showed high concentration of iron where 28% and 40% was found in samples from ground and surface water respectively thus predisposing people to cancer [23]. In addition to the poor water sanitation system and drainage lines, animal and human fecal containing the coli form bacteria are drained in water bodies such as rivers and lakes thus leading to waterborne diseases [16]. In Islamabad and Rawalpindi, analysis done on water samples showed 94% and 34% was contaminated with total coliforms and fecal coliforms, respectively [23]. Furthermore, open defecation is a big problem in most of rural than urban areas in Punjab Provinces, where studies show 25% of the population practice it [24].

Every year, there is about 100 million diarrheal cases and 2.5 million people in Pakistan die of endemic diarrheal disease due presence various pathogens including virus, bacteria, and protozoa in the contaminated ground water supply [25]. Diarrhea accounts for 60% of deaths among infants and children in Pakistan [26]. In addition, everyday 670,000 children miss school due to diseases and lack of proper water sanitation system [22]. Other impurities present in water bodies include trace elements/heavy metals including Zinc, Manganese and copper, as well as pesticides [23].

The most common waterborne diseases are cholera, dysentery, hepatitis, typhoid, giardiasis, intestinal worms, diarrhea, cryptosporidium infections, and gastroenteritis [27]. Most of the water borne diseases are most prevalent in rural areas, especially Pakistan's southern province of Sindh Karachi, and Hyderabad [28,29]. In Islamabad, Pakistan, hepatitis E is most prevalent due to the use of untreated water [30]. In addition, contaminated water usually contains other pathogens such as *E. coli* (51%), Giardia lamblia(27.66%), Enterobacter(64%), Salmonella, Cyclosporin coetaneities, and Clostridium [[31], [32], [33], [34]]. In addition, Naegleria fowleri infections are common in Karachi, in 2019 there were 96 confirmed cases (92% males, 8% females) [35]. Conclusively, these diseases cause harmful effects and unfortunately most people are unaware of the potential risks of using unsafe water for domestic uses such as drinking.

## 6 Preventative techniques and treatment

Some of the major causes of water contamination are unprotected water sources, lack of sanitary practice, unsafe domestic water usage and insufficient management of water treatment. Thus, to further prevent water contamination and improve the quality, governments should increase wastewater treatment plants as well as revamp the existing ones. Amendments in the sewerage system are necessary to prevent sewerage waste from leaking into the drinking water. Additionally it is important that awareness campaigns should be carried out in order to educate the mass on importance of water sanitation along with methods of maintenance [36]. Certain interventions are put in place in order to prevent waterborne diseases such as addressing misconceptions and social customs. WASH education campaigns have successfully been held in attempts to target disease prevention behavior along with hygiene practice within individuals. Keeping in mind the various languages and cultures within Pakistan, education should be delivered in a culturally sensitive manner with respect to the languages and literacy of the communities.

In order to maintain baseline water quality, routine check-ups of the chlorine levels in house hold waters should be performed [37]. Chlorine tablets are administered as water disinfectants and are often efficiently used to kill pathogens present in the water. This method is accepted worldwide due to low-cost and easy application. A study conducted in Pakistan showed a greater than 95% presence of bacteriological contamination [38]. The samples collected from both surface and ground water detected diseases such as Cholera, Typhoid fever and Hepatitis A & E which result in severe prolonged diarrhea which leads to dehydration [36]. Due to severity of the diseases, an addition method of treating water in Pakistan is the application of polymer technology. Through this method suspended solids are coagulated and form floc which are curds of solid material. Cationic polymers such as polylysine result in cell membrane lysis of pathogens by causing leakage of cell components. Low cost of cationic polymers makes it an economical technique of water treatment [39]. Thus, aims at the government level in order to improve water sanitation could focus on surveillance of the water quality, endorsement of water treatment technologies which are low in cost alongside development of more water treatment plants [40].

A community-based survey in the city of Karachi consisting of 379 participants revealed that 89.40% of the participants use the boiling method to treat their water, whereas 7.28% use non-specified methods [41]. In addition, 1.99% use purifiers for water treatment and 1.33% use chemical methods [41]. Reverse osmosis (RO) technology is a popular means to decrease waterborne diseases. This pressure-driven method works by removing organisms from the water by a semipermeable membrane. The filtration of contaminants results in clean drinkable water. Since this particular method requires a great amount of energy and it fails to eliminate volatile organic compounds, an alternative known as Carbon nanotubes (CNTs) are used to treat water. These nanotubes have a hollow structure which is hydrophobic whereas the ends are hydrophilic. This eases the movement of water molecules through the tubes. The membranes of the CNTs have antimicrobial and cytotoxic effects which disrupt the cell membranes of the pathogens. This method has preferable qualities such as ecofriendly, low cost and increased recyclability [36].

The Multiple tube Fermentation technique (MTF) is used to determine the presence of coliform groups in water. This method is composed of three stages, a presumptive stage in which the water sample is placed in multiple test tubes along with a nutrient broth which are then incubated for approximately 48 h. The formation of gas results in a positive presumptive test. Since both coliform and non-coliform bacteria produce gas, a second confirmatory test is performed to specify the organism. Finally, the completed test is performed on the previously positive subjects to further identify the organism. A simpler approach towards detecting fecal contamination in water is the hydrogen sulfide test. Coliform bacteria produce hydrogen sulfide which reacts with iron thereby producing a black iron sulfide precipitate. Water samples are incubated within an iron rich growth medium for 12–18 h. Production of a black color reveals the presence of hydrogen sulfide producing organisms. Microarrays are another promising means to detect waterborne pathogens by simultaneously detecting numerous genes. This method is specifically used to detect *E. coli* virulence and virulence related genes [42]. All preventative and treatment methods have been summarized in a table below.

The National Environmental Action Plan (NEAP) in Pakistan purposed programs such as the clean drinking water program which consisted of two phases, the first phase being installation of plants in various districts and the second phase in which infiltration plants would be involved in union councils along with villages. Within Pakistan there have been certain policies which aim to improve sanitation by developing flush latrines within homes in urban areas and high-density rural areas. This would transfer the waste to a sewerage treatment facility. Ventilated pit privies or pour flush latrines would be placed within homes in low-density rural settlements. This would transfer the waste to a wastewater disposal or collection system. These measures would certify a defecation free environment thus encouraging hygienic practices [40]. Table 1 shows prevention Techniques/Treatments and their effectiveness.

**Table 1:** The preventative techniques used in Pakistan and their effectiveness are summarized in the table below.

Preventative Technique/Treatment	Effectiveness
Chlorine Tablets	Kills low cost, Easily Applicable, widely distributed
Polymer Technology	Tested to remove around 65% of sludge, low cost, not easily applicable, has not been widely distributed.
Reverse Osmosis	Filtration leads to drinkable water, cost-effective, high-energy consumption, does not eliminate organic contaminants
Carbon Nanotubes	Antimicrobial action, low cost, low-energy consumption, recyclable
Multiple Tube Fermentation	Coliform detection, time effective (48 h)
Hydrogen sulfide test	Fecal matter detection, less time effective (12–18 h)
Microarrays	*E. coli* and virulence related gene testing, widely used

## 7 Recommendations and future insights

Globally many programs have been introduced to monitor the water sanitation conditions across countries, especially in the low and middle-class countries as it has been stated that nearly 2 billion people drink water that is contaminated with feces. These programs include the joint monitoring program (JMP) and the Sustainability Development Goals 6 which include the 6 aims to ultimately ‘Ensure availability and sustainable management of water and sanitation for all’ by 2030 [6].

Relative to the rest of the world, Pakistan which is ranked in the top 10 of countries with the highest populations living without access to safe water, is in drastic need of improving its water sanitation practices as well as its drinking water standards throughout its cities and rural areas [43]. To work on water sanitation, key initiatives need to be taken place not only at the government level but also simultaneously at the individual level. If applied correctly, the death and diseases caused by water-borne diseases can be significantly reduced by maintaining adequate hygiene.

At the government level, the legislative approach should be used and water authorities like WASA, and the Water and Sanitation Agency should be held responsible for the quality of drinking water [4]. Industrial waste and commercial waste should not be dumped directly into freshwater resources as they can be hazardous to human health, as increased levels of nitrates have been reported to cause blue baby syndrome in bottle-fed children. It is essential for the government to go after companies and individuals which actively contribute to the waste being dumped as such by heavily fining them, holding them responsible for disposing of the waste, as well as potential prison time. This will deter additional people from committing the same act, have those which already done so clean up the present waste, as well as provide an opportunity to use the collected fines to build treatment facilities to further improve water sanitation. Water sanitation and water-borne disease are major health concerns so more investment at the government level should be done to work on projects to improve the quality of drinking water. However, although it is true that the country has invested in creating new treatment plants to do just that, simply building these facilities is not enough. According to an official of the Karachi Water and Sewage Board (KWSB), once these facilities are built and installed by foreign companies, they immediately leave without any guidance on running and maintaining the treatment plants. As a result, the technicians present become underqualified and are unable to maintain the facilities eventually leading to them running under capacity or even potentially collapsing quicker than initially estimated [44]. Therefore, it's imperative for the government to not only build the facilities, but also provide the training and technical knowledge to run and maintain them as well.

Along with the government, the private sector should be encouraged as well to work on water sanitation projects as it can help in water problems in the long term [22]. For example, in terms of water sanitation and hygiene, several campaigns on the level of both public and private schools as well as colleges should be conducted so that the younger generations can learn and practice different hygiene techniques. This is effective as not only will the students be taught on mass about the practices and benefits of water sanitation and proper hygiene, but the same students will be able to teach their own family members back at home thus making the students their own agents of change. Additionally, the use of mass media can be very beneficial in the long term for raising awareness regarding water sanitation which should be done simultaneously by spreading this information across various social media platforms and by employing social media influencers who can reach a wide audience.

Finally on an individual level, practicing various water sanitation technique can help improve the water quality. This is especially important and true in rural areas where public health campaigns can be useful in terms of reinforcing the quality standard. One reason for this is that rural areas are vastly underfunded and undereducated compared to their counterparts living in the city. Furthermore, it is in rural areas which have the biggest problem with open defecation, poor hygiene, and a lack of proper waste management. This is significant as a majority of water in rural settings comes from ground water with over 50% of village households drinking water source coming from hand pumps [24]. This supplemented with the fact that the nearly no investment goes to the management of fecal sludge and wastewater leads to drinking water and soil contamination for the inhabitants of the village. Therefore, not only should actions be made to improve the water sanitation capabilities across the country, but also there should be plans made to invest in the effective management and disposal of fecal matter and wastewater. This being implemented with proper hygiene practices will lead to the water quality in Pakistan improving in both its cities and rural areas.

## 8 Conclusion

This paper focused on water sanitation and disease prevalence in Pakistan and the current methods and programs that are being implemented to improve water sanitation. With water-borne disease on the rise and the adverse effects of poor water sanitation on human health, immediate steps need to be taken both on individual and government levels. Globally accepted programs of water sanitation should be enforced across the country and new programs of water sanitation that are cost-effective should be introduced.

## Ethical approval

None.

## Please state any sources of funding for your research

None.

## Authors’ contribution

All authors contributed equally in study concept or design, data collection, data analysis or interpretation and writing the paper.

## Registration of research studies

Name of the registry:
Unique Identifying number or registration ID:
Hyperlink to your specific registration (must be publicly accessible and will be checked):

## Guarantor

N/A.

## Consent

N/A.

## conflicts of interest

None declared.
